# A Treatise on the Acute, Infectious Exanthemata

**Published:** 1902-02

**Authors:** 


### BOOK REVIEWS.

A Treatise on the Acute, Infectious Exanthemata. Including Variola, Rubeola, Scarlatina, Rubella, Varicella and Vaccinia, with especial reference to Diagnosis and Treatment. By William Thomas Corlett, M. D , L. R. C. P., London, Professor of Dermatology and Syphilology in Western Reserve University; Physician for Diseases of the Skin to Lakeside Hospital; Consulting Dermatologist to Charity Hospital, St. Alexis Hospital, and the City Hospital, Cleveland. Illustrated in 12 Colored Plates, 28 Half-tone Plates from Life and 2 Engravings. Pages viii-392. Philadelphia: F. A. Davis Company, Publishers, 1914-16 Cherry Street. [Price, Extra Cloth, $4,00 net, Delivered.

This book, the author tells us, is especially intended to benefit young practitioners who are often confronted by their first case of exanthemata after having fairly begun the practice of medicine, and are subject to chagrin and mortification when the invincible old woman readily tells them from what special form of the ailment the patient is suffering. One would almost conjecture that the author himself had passed through this particular experience, judging from the opening sentence of his preface and that, after many years of patient and careful study, he had been able to prepare a work whose value to the medical profession—especially to its younger members—it is simply impossible to compute.

The initiatory portion of the volume deals with the early history of exanthemata, covering twenty-seven pages. In tracing the history of smallpox the author reviews the efforts that have been made to assign to it a prehistoric antiquity, on the part of those who assume to believe that pestilence and plague are used synonymously with that disease and other eruptive fevers, and by whom, when smallpox is first spoken of, it was not characterised as anything new—all of which leads the author to suppose that the disease is really older than the records show. A résumé is given both of the history and of the geographical distribution of this disease in each country, as it existed during prehistoric times. The description of smallpox credited to Hippocrates is meager and may fairly be considered as questionable.

The history of smallpox in the Americas dates from 1517, when both it and measles were carried to the Island of St. Domingo. Smallpox prevailed in China and Hindoostan from remote antiquity, but did not extend through the Western Hemisphere until later. In this connection a plate constituting a religious dramatic representation of the power of the Hindoo Goddess of smallpox possesses genuine interest. The second chapter opens with a consideration of variola, and presents an intelligent conception, modern in thought, of the different forms of smallpox, with logical reasons for the predominance of the irregular or middle forms, as found in the present outbreak in the United States; showing, also, that the disease, which frequently varies in manifestations, is but a contingent of the same condition—a fact which may be considered unanswerable. Of course, the enforcement of vaccination on all well-regulated cities accounts for the fact that the number of malignant cases has become largely diminished.

The literature of the last few years relating to the manifestations of smallpox in this country has been abundant, but much of it misleading and pernicious. The volume under consideration brings the discussion of the subject up to the present day, without giving much attention to the ignoble army of writers who are laboring to establish a new disease theory by way of cloaking their pitiful ignorance and covering their unfortunate mistakes; some calling it chickenpox, others, a new disease.

Dr. Corlett has given a concise and systematic interpretation of this subject, as he is well qualified to do both by experience and native ability. He lays special stress upon diagnosis, which, in typical cases can readily be made, although attended by more or less uncertainty at the beginning. He calls attention to the fact that no single feature of a dermapathy is absolutely pathognomonic of smallpox, and declares that physicians not uncommonly hold their patients for an unnecessarily long time waiting for the assertion of all the conventional symptoms. He portrays the physiognomy of this disease in the fewest words possible, and in differential diagnosis is most exact, although he makes one statement in relation to chickenpox which is calculated to create surprise—namely, that he himself has never encountered a case of chickenpox arising after puberty. The reviewer distinctly recalls four cases of chickenpox that appeared after the age of twenty-five. Twenty illustrations relating to smallpox accompany the text, of which five are colored plates. The latter were made by a well-recognised medical artist, and show much painstaking and care, and are, for the most part, quite true to nature, although they leave something to be desired in the matter of coloring and flesh tints. The selection of cases for photographic reproductions has been made with a view of illustrating the present epidemical character of the disease. The reproductions themselves, all the usual drawbacks and hindrances being considered, are remarkably good.

The subject of vaccine occupies twenty-two pages, and is exhaustively treated. The author has paid proper attention to anomalous vaccine, and the complications and sequelæ. We might reasonably have expected a few more plates, illustrating various forms of eruption following vaccination; a lack probably accounted for by the present rarity of this result, although plates XXI. and XXII. are exceptionally good.

So much has been said about varicella that it is refreshing to see a correct photographic reproduction of this common disease. In order to get a just idea of what a typical case looks like, one needs to compare this picture of varicella plate XXIX. with variola plate XIX; the two diseases having so much in common. Plate XXVII. and XXVIII. are open to criticism on account of their unnatural redness, but all must concede that the pictures give a very good idea of the general character of the disease. In the description proper, stress is laid on diagnosis and treatment.

The work is concluded with a description of scarlatina, rubeola and rubella which contains much that will be found in other standard treatises on general medicine; yet for admirable arrangement of the material, the omission of useless details, the clear mode of expression and a certain original and forcible method of treating his subjects, especially in the matter of diagnosis, the author deserves high praise. While the work represents the author's individual views and opinions he has not neglected to draw upon the observations and researches of men in general medicine, and has successfully combined them with his own. There are photographs and colored plates scattered through the text which will be of great assistance to practitioners as they are well executed, illustrate features in common cases and accent the points of differential diagnosis.

After a careful examination of Dr. Corlett's book, we wish to congratulate him on giving to the public something that was much needed. Especial prominence is most properly given in the work to the consideration of the disease (smallpox) most prevalent in the United States. The exposition of the principles and practices in the diagnosis of acute exanthemata is both valuable and creditable.

The book is printed in clear, readable type, upon paper of superior quality and presents a handsome and inviting appearance.—G. W. W.

